# Physicians' Perspectives on Barriers to Multiple Sclerosis Care in the United Arab Emirates: A Survey Study

**DOI:** 10.7759/cureus.77281

**Published:** 2025-01-11

**Authors:** Ruqqia Mir, Lev Brylev, Sabah Zahoorudin, Rene A Rivero Jimenez, Rosario Servano, Yandy M Castillo-Aleman, Maysoon M Al Karam, Yendry Ventura-Carmenate, Fatema Al-Kaabi

**Affiliations:** 1 Neurology, Yas Clinic Khalifa City, Abu Dhabi Stem Cells Centre, Abu Dhabi, ARE; 2 Operations, Yas Clinic Khalifa City, Abu Dhabi Stem Cells Centre, Abu Dhabi, ARE; 3 Immunology, Stem Cells Processing Laboratory, Yas Clinic Khalifa City, Abu Dhabi Stem Cells Centre, Abu Dhabi, ARE; 4 Neurology, Abu Dhabi Stem Cells Centre, Abu Dhabi, ARE; 5 Immunology, Yas Clinic Khalifa City, Abu Dhabi Stem Cells Centre, Abu Dhabi, ARE; 6 Ophthalmology, Yas Clinic Khalifa City, Abu Dhabi Stem Cells Centre, Abu Dhabi, ARE; 7 Hematology, Abu Dhabi Stem Cells Centre, Abu Dhabi, ARE

**Keywords:** clinical features, epidemiology, healthcare quality, multiple sclerosis, survey

## Abstract

Introduction: Multiple Sclerosis care in the United Arab Emirates faces several barriers impacting patient outcomes and healthcare delivery. This study aims to identify and analyze these barriers from the perspective of neurologists.

Methods: In February 2024, a survey was conducted targeting neurologists involved in Multiple Sclerosis management in Abu Dhabi. The survey included questions on demographics, regularly measured clinical variables, perceived barriers, and contributing factors. A total of 21 neurologists responded, and data were analyzed to identify prevalent issues and correlations.

Results: Among the 21 respondents, 15 (71.4%) were male, and 15 (71.4%) were from private institutions. Most neurologists regularly advised on vitamin D level monitoring (16, 76.2%) and psychological support (15, 71.4%). Screening for disease-modifying treatment side effects, Expanded Disability Status Scale assessments, and magnetic resonance imaging comparisons were practiced, but improvement is needed, especially in private settings (10, 47.6%). Significant barriers identified included the high cost of medications (11, 52.4%), insurance-related issues (10, 47.6%), and socioeconomic factors (9, 42.9%). Physician-related barriers, such as time constraints (8, 38.1%) and lack of specialist nursing support (4, 19.0%), were also prominent. System-related barriers included insurance provider policies (13, 61.9%) and gaps in clinical guideline coverage (11, 52.4%). Gender differences in perceived barriers were significant (p=0.016), while age was not (p=0.147).

Conclusions: This study highlights critical barriers to Multiple Sclerosis care in Abu Dhabi, emphasizing the need for policy changes, improved insurance coverage, and targeted support for patients and healthcare providers. Addressing these barriers is essential for enhancing the region's quality of Multiple Sclerosis care and patient outcomes.

## Introduction

Multiple sclerosis (MS) is a chronic neurological condition that significantly impacts patients' quality of life due to its unpredictable course and progressive nature. Effective management of MS is crucial to alleviate symptoms and improve patient outcomes, adhering to global standards and guidelines [1-3]. In the United Arab Emirates (UAE), the healthcare system has made strides in providing care for MS patients, yet unique regional challenges exist. Recent initiatives aim to enhance MS care, but comprehensive data on current practices and infrastructure remain limited [2,3]. Understanding these practices is essential to identifying areas for improvement.

MS care globally faces numerous challenges, including limited access to specialized treatments and variability in care standards. In the UAE, these issues are compounded by region-specific factors such as healthcare infrastructure and patient demographics [3,4]. Previous studies have highlighted these obstacles, emphasizing the need for targeted interventions. Despite the challenges identified, there is a notable gap in the literature concerning the specific barriers to MS care in the UAE from the perspective of healthcare providers. Current research primarily focuses on patient outcomes and clinical data, leaving a void in understanding physicians' viewpoints [4-7]. Addressing this gap is crucial for comprehensive care improvement.

This study aims to fill the gap by directly surveying physicians involved in MS care to understand the barriers they face. By gathering insights from healthcare providers, the study seeks to identify factors contributing to these barriers and propose solutions to enhance MS care practices. The survey focuses on key topics such as resource availability, treatment accessibility, and administrative support. It aims to provide a detailed understanding of the challenges physicians encounter and how these impact their ability to deliver optimal care to MS patients.

The findings of this study will be valuable for policymakers, healthcare providers, and patients. They will inform future research and healthcare strategies, contributing to the overall improvement of MS care in the UAE and potentially serving as a model for other regions.

## Materials and methods

This cross-sectional survey study was conducted in February 2024 to gather insights from neurologists on the barriers to MS care in Abu Dhabi and across the UAE. Participation was voluntary, and electronic informed consent was obtained from all respondents before completing the survey. All data were collected and analyzed anonymously, with no personally identifiable information recorded, ensuring participant confidentiality.

Sample size and participants

A purposive sampling technique was employed. Invitations were sent via email to 120 neurologists practicing in Abu Dhabi and affiliated regions of the UAE. The neurologists were identified through professional networks and local neurology societies. The sample size (n=120) was chosen based on the estimated number of practicing neurologists involved in MS management in the region and practical considerations regarding survey distribution and response rates. Of the 120 invited neurologists, 21 completed and returned the survey, resulting in a response rate of 17.5%.

Inclusion and exclusion criteria

Inclusion criteria were (1) licensed neurologists currently practicing in the UAE; (2) direct involvement in the diagnosis, management, or follow-up of patients with MS; and (3) willingness to provide informed consent and participate in the survey. Exclusion criteria were (1) healthcare professionals not specializing in neurology, (2) neurologists not involved in MS patient care, and (3) those who declined to provide consent or did not complete the survey in full.

Survey instrument and data collection

The questionnaire used in this study was adapted from a previously published instrument examining global barriers to MS care [8]. The original questionnaire was described in detail by Solomon et al. [8] and was modified slightly to reflect regional healthcare structures and resources available in the UAE (Appendix). The adapted survey covered demographics, clinical management practices, perceived barriers, physician-related factors, and healthcare system-related challenges.

A multidisciplinary team of neurologists and quality improvement specialists reviewed and refined the adapted instrument to ensure its relevance and clarity. It comprised multiple sections. The first section focused on demographics and practice characteristics, collecting information such as the respondents’ age range, gender, place of practice (private, government, or semi-government), and knowledge of quality improvement tools. The second section addressed clinical management and monitoring, with questions about variables routinely measured every 6-12 months, including vitamin D levels, screening for disease-modifying treatment side effects, and the use of the Expanded Disability Status Scale (EDSS) or magnetic resonance imaging (MRI) comparisons. Another section explored patient advice, focusing on recommendations provided to MS patients, such as smoking cessation, weight management, psychological support, and monitoring blood pressure and glycemic control.

The survey also investigated perceived barriers and contributing factors, using open-ended and multiple-choice items to identify challenges related to patients, physicians, and the healthcare system. Respondents ranked these barriers and highlighted factors contributing to difficulties in MS care delivery. Additionally, the survey assessed the role of social tools and interventions, aiming to identify community or societal resources that could best improve MS care. The survey was distributed electronically via a secure online platform, with two reminder emails sent at one-week intervals to enhance response rates. Responses were automatically recorded in Microsoft Excel to ensure accuracy and minimize transcription errors.

Statistical analysis

Statistical analysis was performed using Python 3.12.0 with the matplotlib library for data visualization. After data extraction into a CSV format, Python scripts were used to compute frequencies, percentages, and cross-tabulations. Descriptive statistics were generated to summarize participant demographics, practice characteristics, and perceived barriers. Visualizations, including bar charts and stacked plots, were created using matplotlib to illustrate trends, distributions, and comparisons across different physician subgroups, thus enhancing interpretability and facilitating the identification of key patterns in the survey responses.

## Results

A total of 120 neurologists were invited to complete the survey, and 21 responded (17.5%). Of these 21 respondents, 15 (71.4%) were male and 6 (28.6%) were female. The age distribution showed that two (9.5%) were aged 26-35, four (19.0%) were 36-45, seven (33.3%) were 46-55, and five (23.8%) were 56 and above, with three (14.3%) unspecified. Regarding the place of practice, 15 (71.4%) were from private institutions, four (19.0%) from government institutions, and one (4.8%) from semi-government institutions, with one (4.8%) unspecified. A majority, 18 (85.7%), were knowledgeable about quality improvement tools, while three (14.3%) were not. The quality measurement tools used included guidelines from the American Association of Neurologists (2, 9.5%), the Middle East North Africa Committee for Treatment and Research in Multiple Sclerosis (MENACTRIMS) (9, 42.9%), and the National Institute for Health and Care Excellence (NICE) (2, 9.5%) (Table 1) [9-11].

**Table 1:** Demographics summary. MENACTRIMS: Middle East North Africa Committee for Treatment and Research in Multiple Sclerosis; NICE: National Institute for Health and Care Excellence.

Category	Sub-category (age range)	Count (%)
Age	26-35	2 (9.52)
	36-45	4 (19.05)
	46-55	7 (33.33)
	56 and above	5 (23.81)
	Unspecified	3 (14.29)
Gender	Male	15 (71.43)
	Female	6 (28.57)
Place of practice	Private	15 (71.43)
	Semi-government	1 (4.76)
	Government	4 (19.05)
	Unspecified	1 (4.76)
Knowledge of quality improvement tools	Yes	18 (85.71)
	No	3 (14.29)
Quality measurement tool	American Association of Neurologists	2 (9.52)
	MENACTRIMS	9 (42.86)
	NICE guidelines	2 (9.52)

The survey included responses to the question: "Do you advise your patients on any of the following regularly?" The results are summarized in Table 2.

**Table 2:** Summary of patient advice data.

Variable	Count (%)
Weight management	10 (47.62)
Blood pressure management	11 (52.38)
Glycemic monitoring	9 (42.86)
Smoking cessation	14 (66.67)
Vitamin D level monitoring	16 (76.19)
Psychological support	15 (71.43)

The majority of neurologists (16, 76.2%) regularly advise their patients on vitamin D level monitoring, followed closely by psychological support (15, 71.43%) and smoking cessation (14, 66.67%). Blood pressure management (11, 52.38%) and weight management (10, 47.62%) are also common recommendations. While still significant, glycemic monitoring is advised by nine (42.9%) of the respondents. These findings strongly emphasize holistic care approaches that address both physical and psychological aspects of MS management.

The patient's insurance level significantly influences the quality and frequency of checking certain variables, such as vitamin D levels and smoking cessation. Patients with higher insurance coverage are more likely to receive comprehensive monitoring and advice, including regular vitamin D level checks and smoking cessation support. Conversely, those with limited insurance coverage may not have consistent access to these preventive measures, highlighting an inequity in healthcare provision that needs addressing. This disparity underscores the importance of improving insurance policies to ensure equitable healthcare access and quality for all patients.

**Figure 1:**
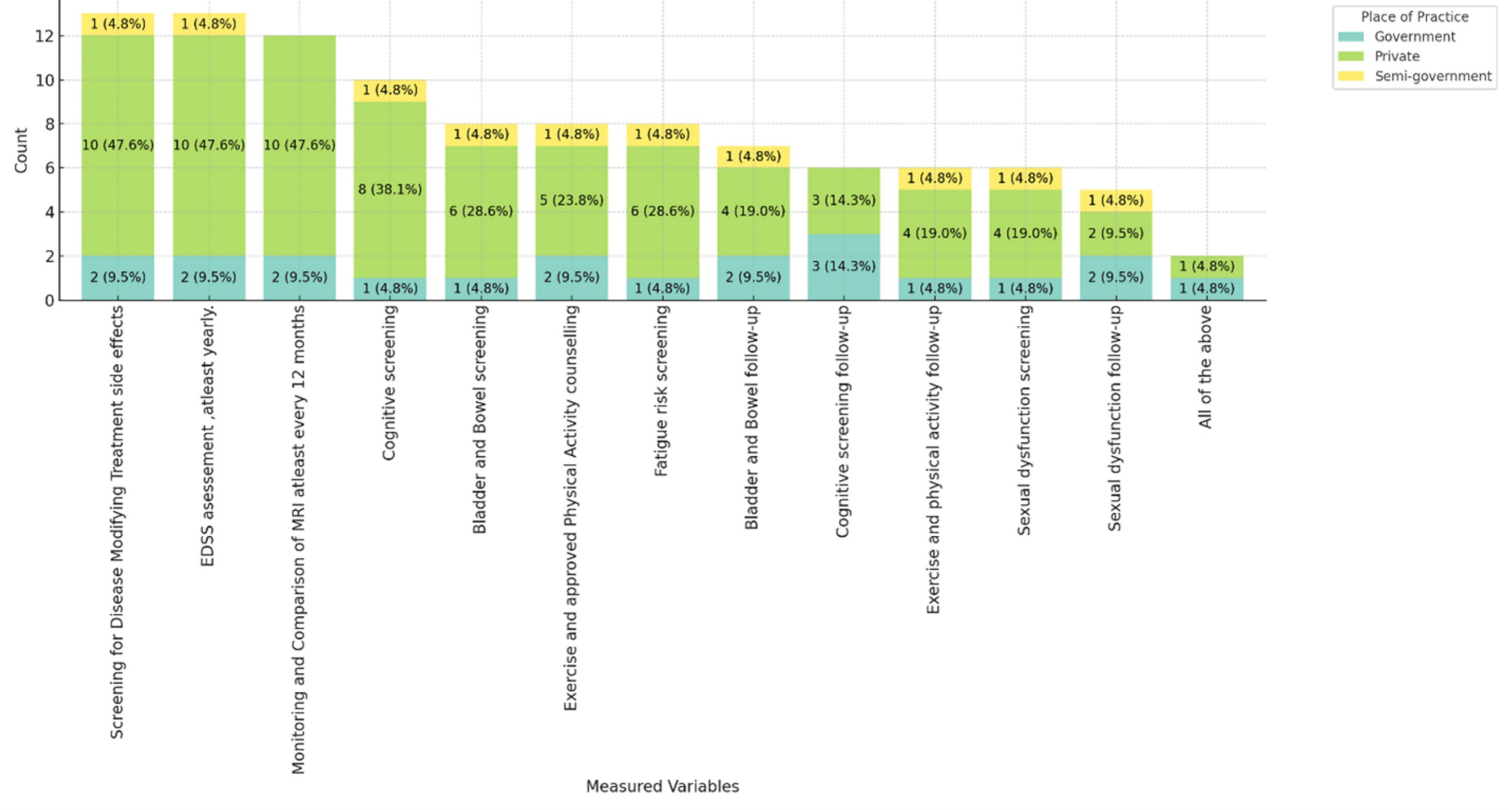
Variables regularly measured in MS care, stacked by place of practice. MS: multiple sclerosis.

Figure 1 illustrates the variables regularly measured in MS care, categorized by place of practice (government, private, and semi-government). Screening for disease-modifying treatment side effects, Expanded Disability Status Scale (EDSS) assessment, and magnetic resonance imaging (MRI) comparisons are predominantly carried out by neurologists in private practices, each reported by 10 (47.6%) of respondents from private settings. Cognitive screening (8, 38.1%) and bladder and bowel screening (6, 28.6%) followed and were primarily conducted in private practices. Semi-government practices show minimal engagement across these variables. Other activities, including physical activity counseling, fatigue risk screening, and various follow-ups, show a varied distribution, with private practices leading in most categories. This chart emphasizes the more extensive engagement in private practices, with government and semi-government practices participating to a lesser extent.

**Figure 2:**
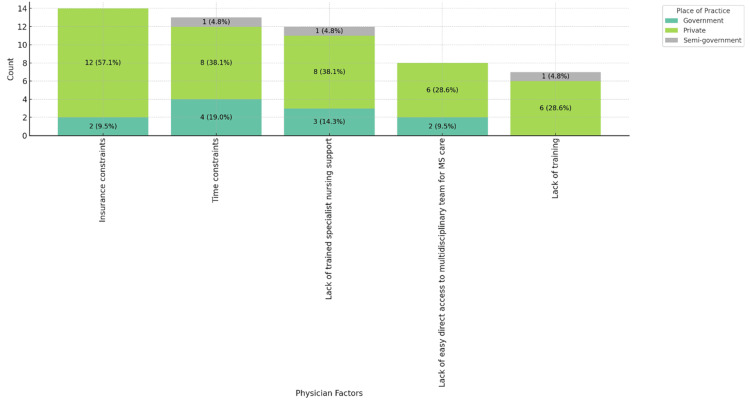
Physician factors contributing to barriers in MS patient care, stacked by place of practice. MS: multiple sclerosis.

Figure 2 shows that insurance constraints (12, 57.1%) and time constraints (8, 38.1%) are the primary barriers to MS patient care, as reported predominantly by private practice physicians.

**Figure 3:**
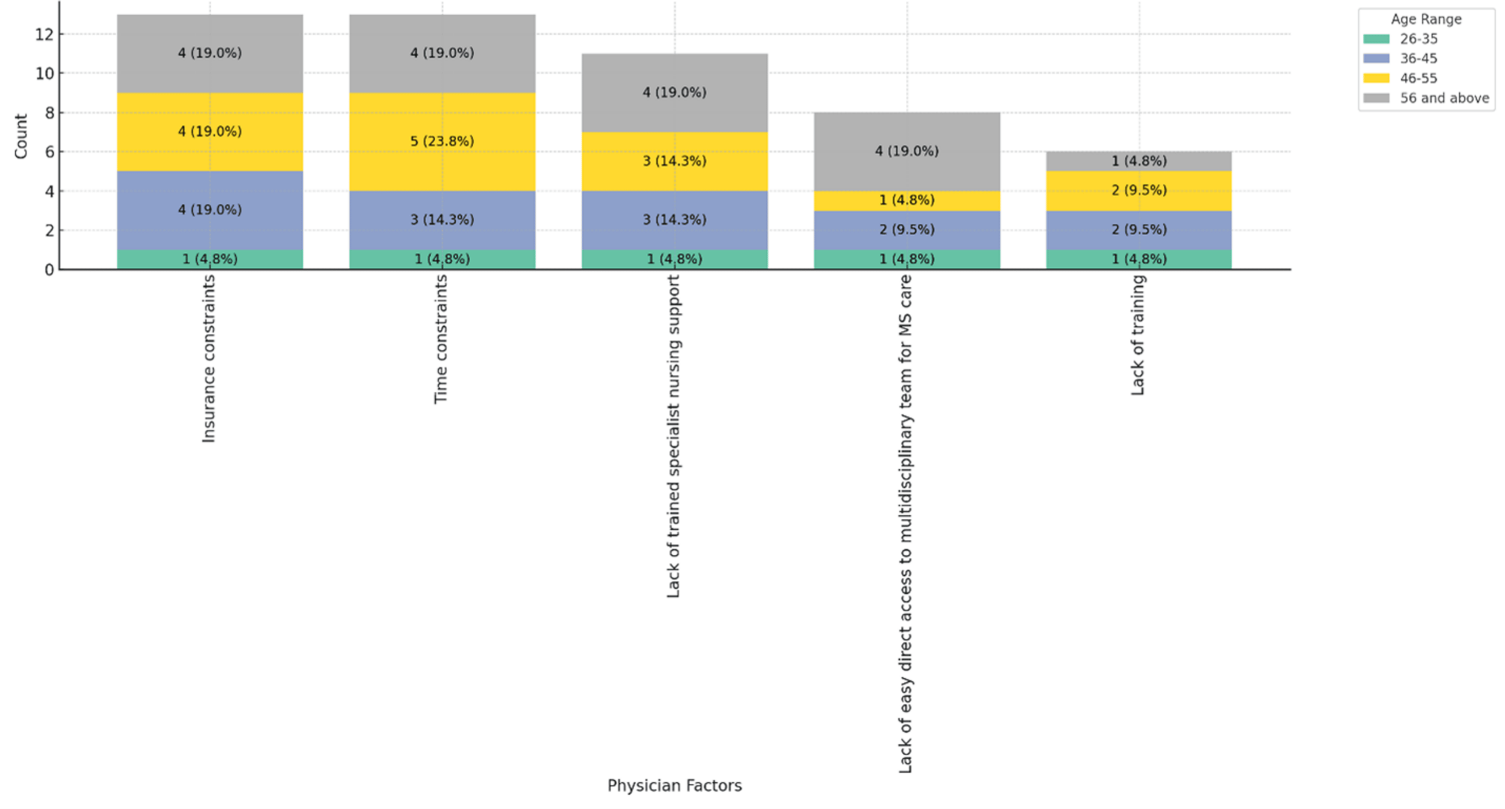
Physician factors contributing to barriers in MS patient care, stacked by age range. MS: multiple sclerosis.

Figure 3 indicates that insurance and time constraints uniformly affect MS patient care across all physician age groups, showing no significant age relation (P = 0.147).

**Figure 4:**
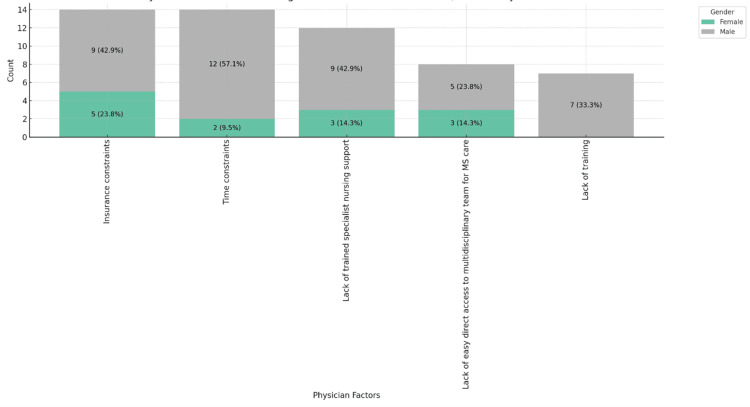
Physician factors contributing to barriers in MS patient care, stacked by place of gender. MS: multiple sclerosis.

Figure 4 reveals gender differences: male physicians report more insurance and time constraints, while female physicians cite insufficient specialist nursing support and access to multidisciplinary teams more often, with a significant gender relation (P = 0.016), suggesting the need for gender-specific strategies.

**Figure 5:**
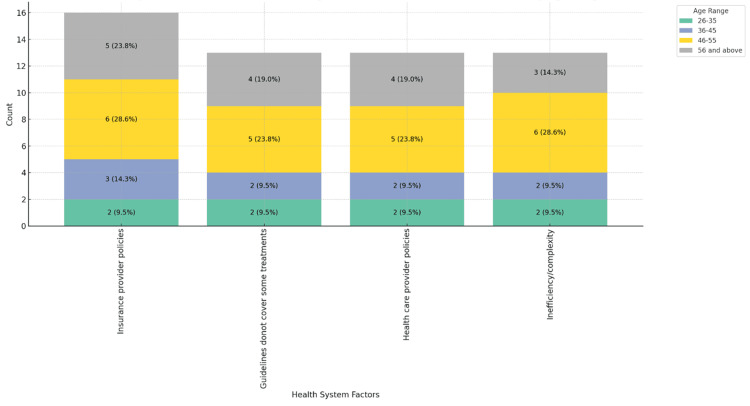
Healthcare system-related factors contributing to barriers in MS patient care, stacked by age range. MS: multiple sclerosis.

**Figure 6:**
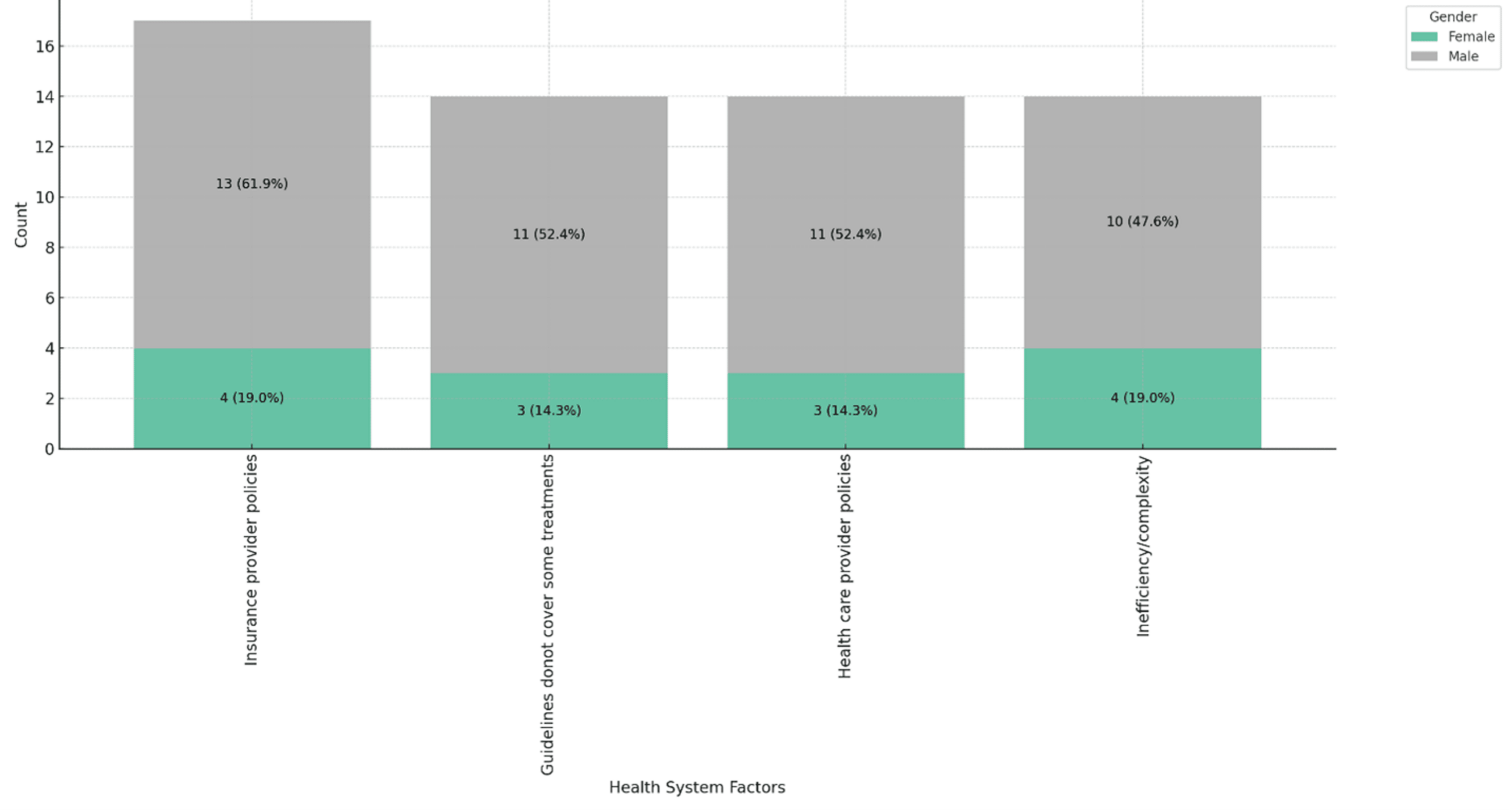
Healthcare system-related factors contributing to barriers in MS patient care, stacked by gender. MS: multiple sclerosis.

**Figure 7:**
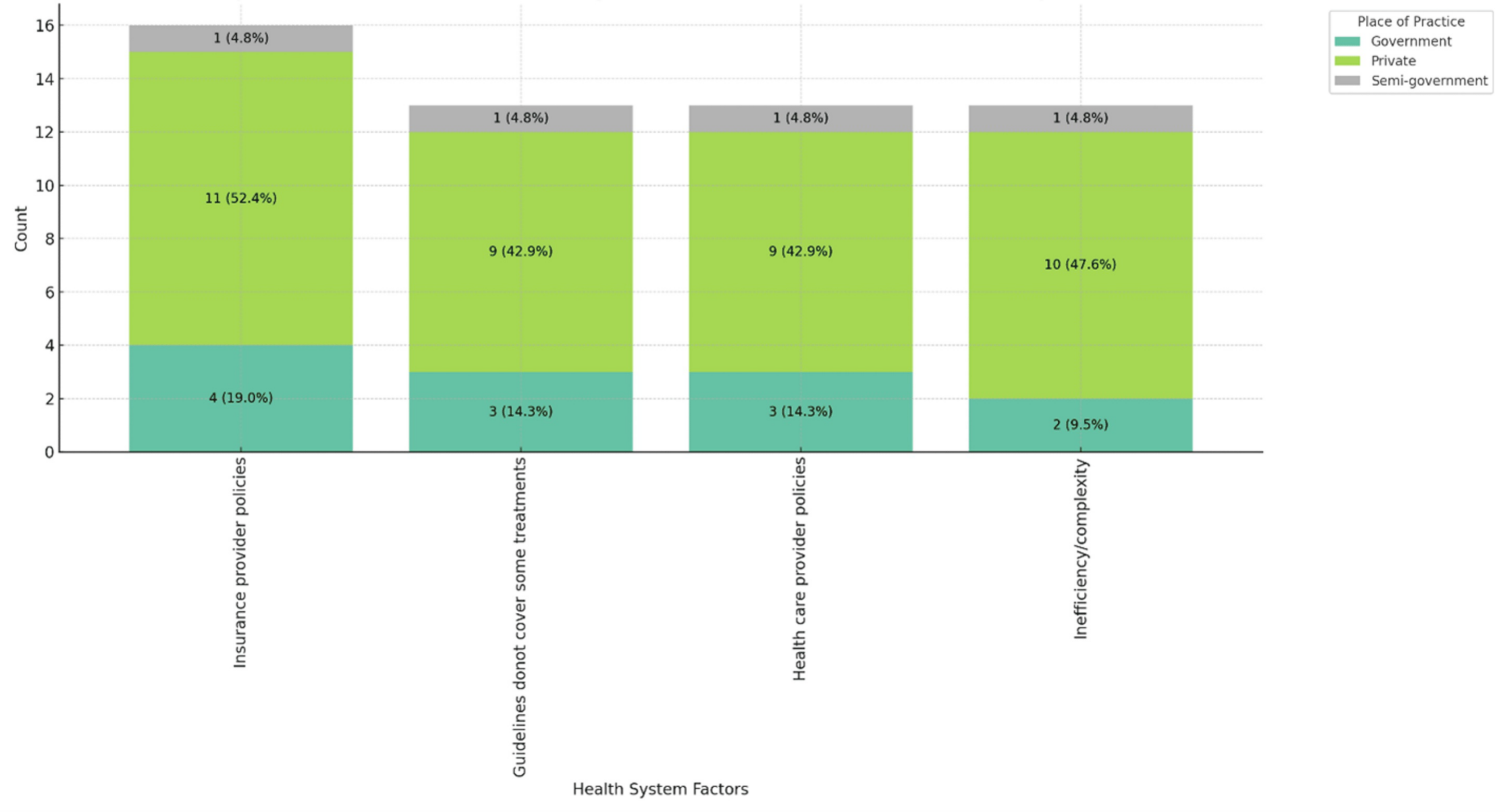
Healthcare system-related factors contributing to barriers in MS patient care, stacked by place of practice. MS: multiple sclerosis.

Figures 5-7 highlight the impact of healthcare system factors on MS patient care. Figure 5 shows insurance provider policies as the main barrier across age groups, especially notable in the 46-55 range, 6 (28.6%). Figure 6 reveals a gender disparity, with 13 (61.9%) male physicians citing these policies as a barrier, compared to 4 (19.0%) female physicians. Figure 7 shows that private practice physicians (11, 52.4%) report these issues more frequently than those in government settings (4, 19.0%).

**Figure 8:**
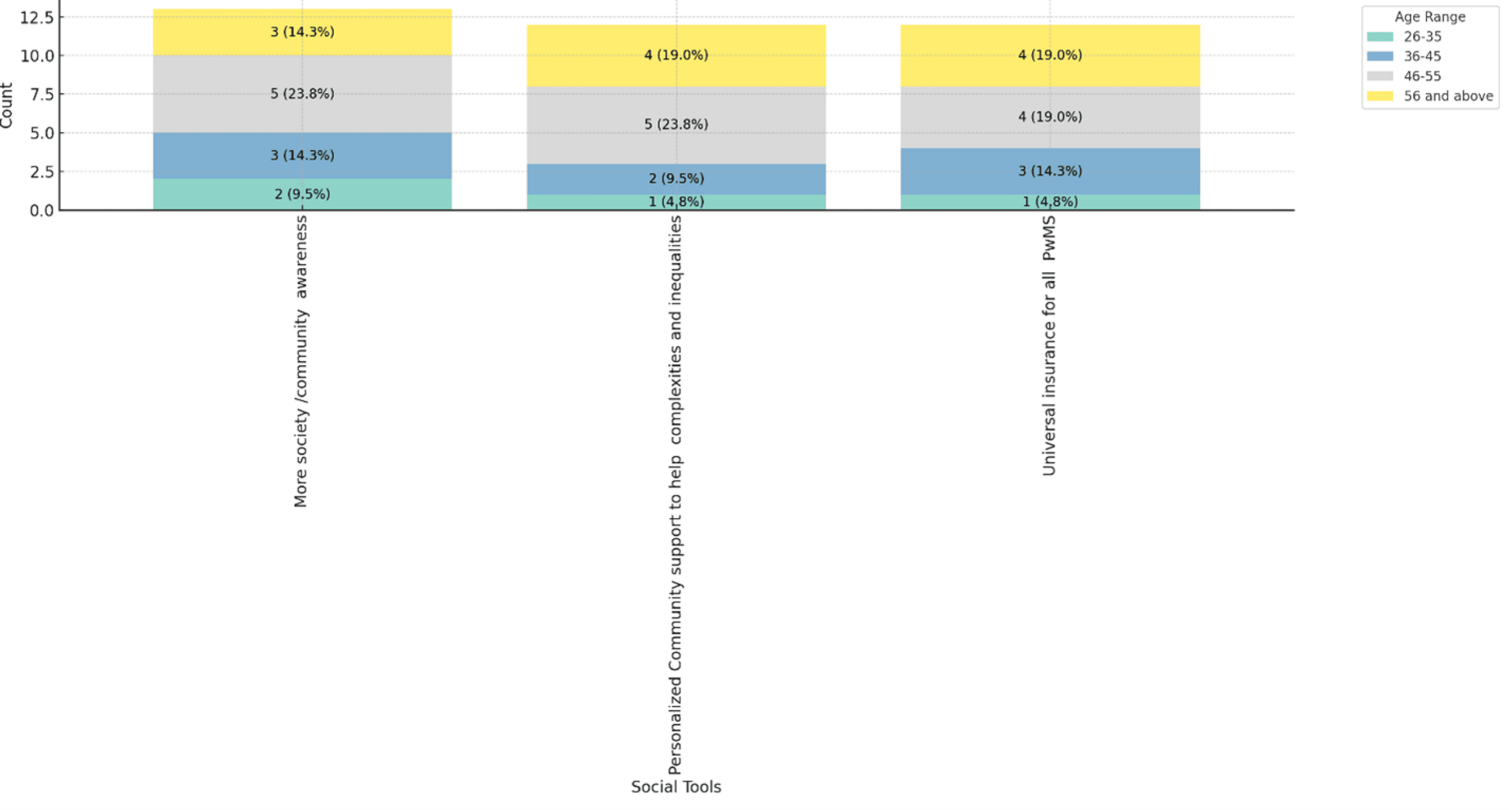
Social tools best for improving MS care in practice, stacked by age range. MS: multiple sclerosis; PwMS: people with multiple sclerosis.

**Figure 9:**
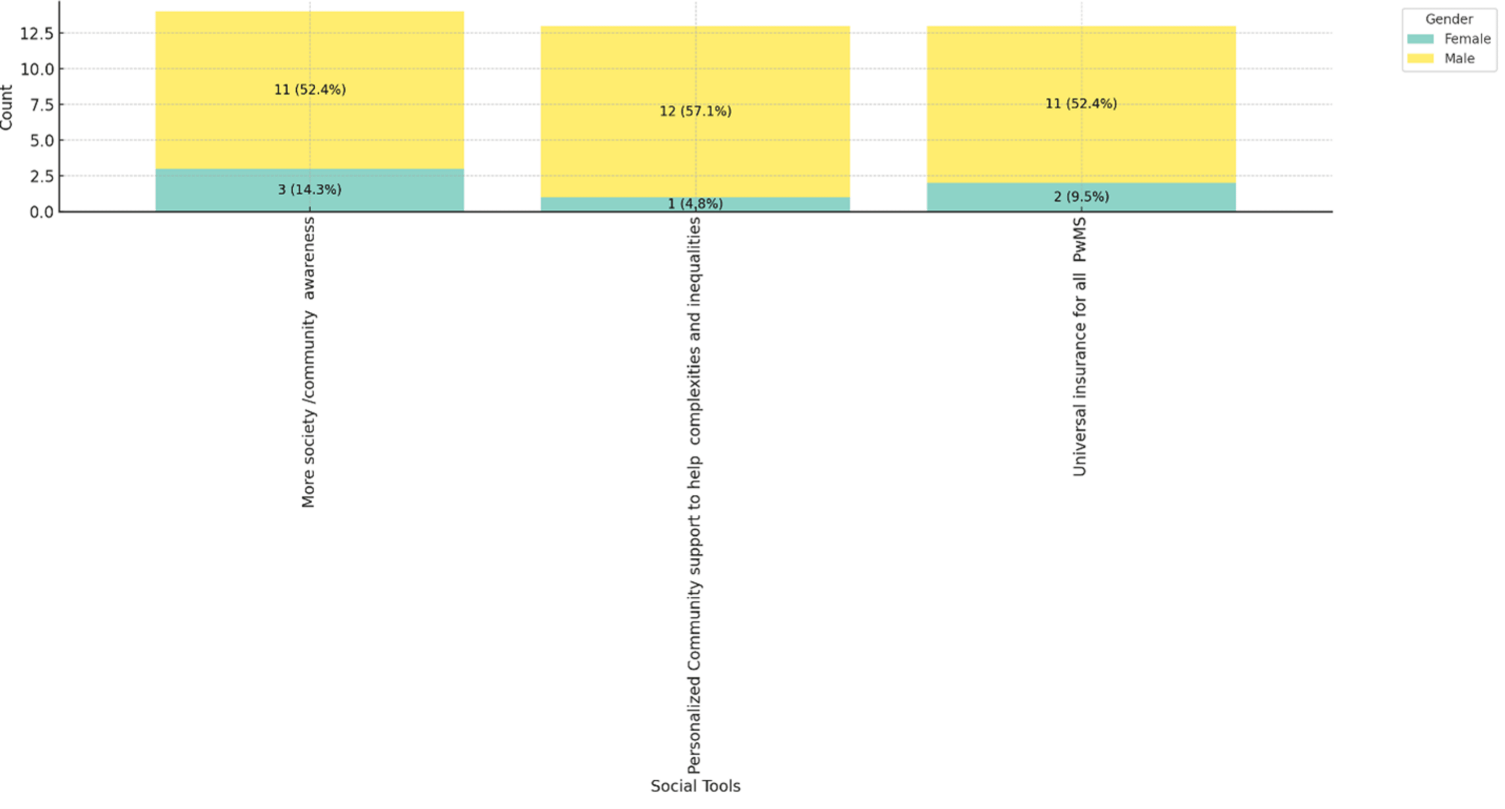
Social tools best for improving MS care in practice, stacked by gender. MS: multiple sclerosis; PwMS: people with multiple sclerosis.

**Figure 10:**
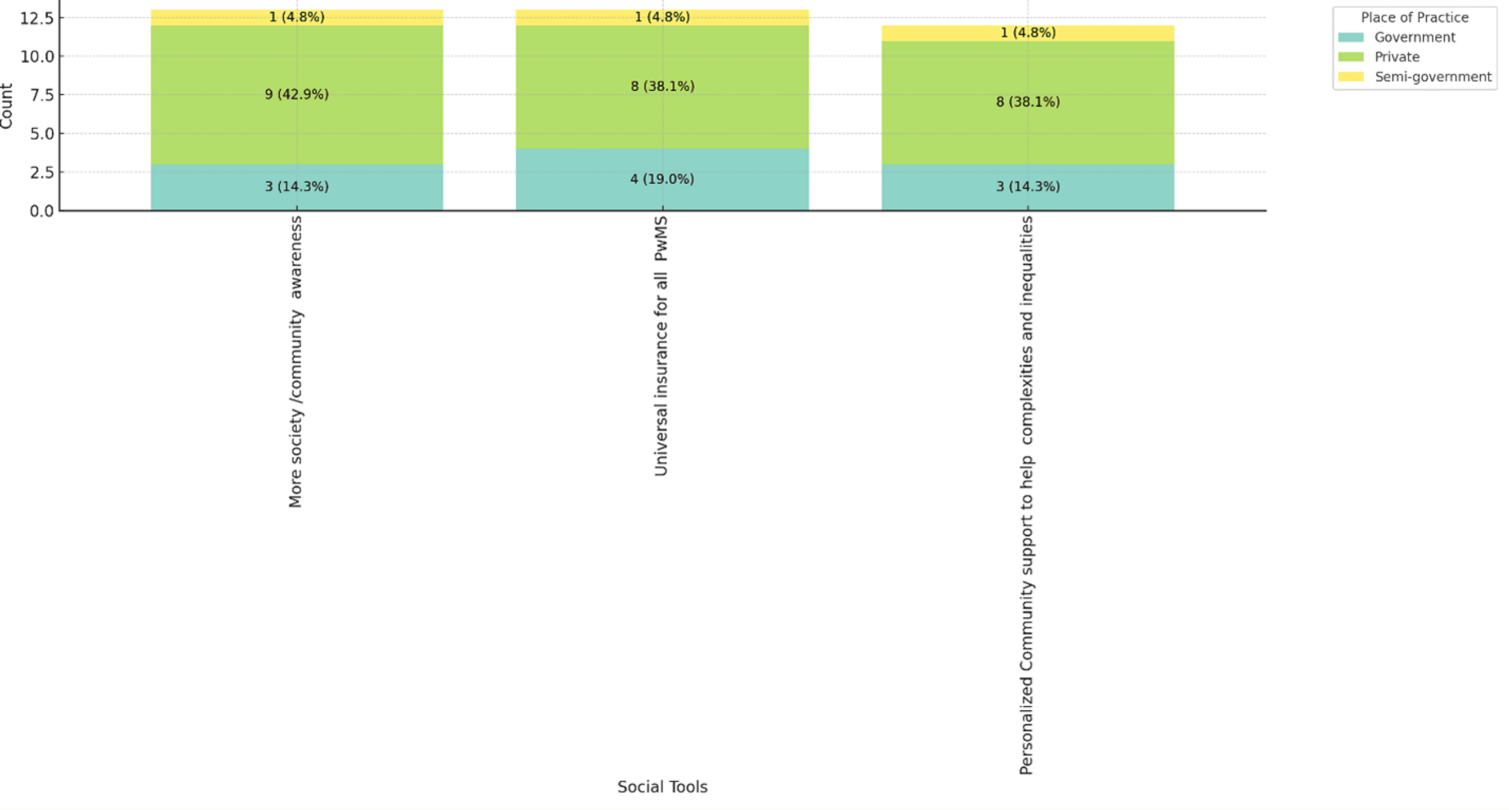
Social tools best for improving MS care in practice, stacked by place of practice. MS: multiple sclerosis; PwMS: people with multiple sclerosis.

Figures 8-10 depict the social tools considered best for improving MS care, categorized by age range, gender, and place of practice. Figure 8 shows that personalized community support and universal insurance for all people with MS are highly valued across all age groups, with more society/community awareness being slightly more favored by respondents aged 46-55, 5 (23.8%). Figure 9 reveals that both male (11, 52.4%) and female (12, 57.1%) respondents prioritize personalized community support and universal insurance equally. Figure 10 indicates that these social tools are consistently favored across different practice settings, with private practice respondents slightly more inclined towards personalized community support (8, 38.1%). These figures highlight the universal recognition of the importance of community support and comprehensive insurance in improving MS care across various demographics and practice environments.

**Figure 11:**
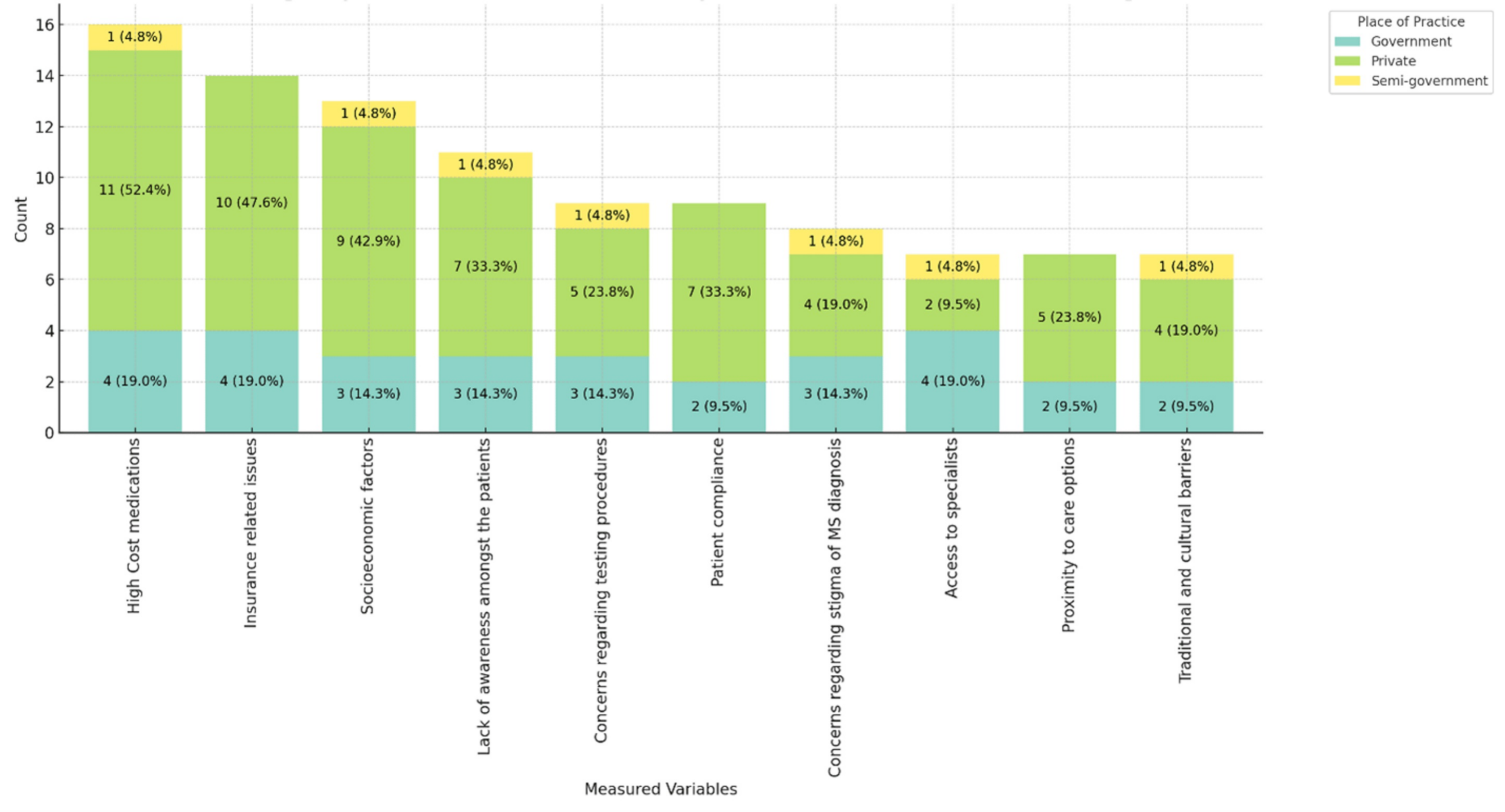
Barriers physicians believe affect the care of MS patients in their practice, stacked by place of practice. MS: multiple sclerosis.

**Figure 12:**
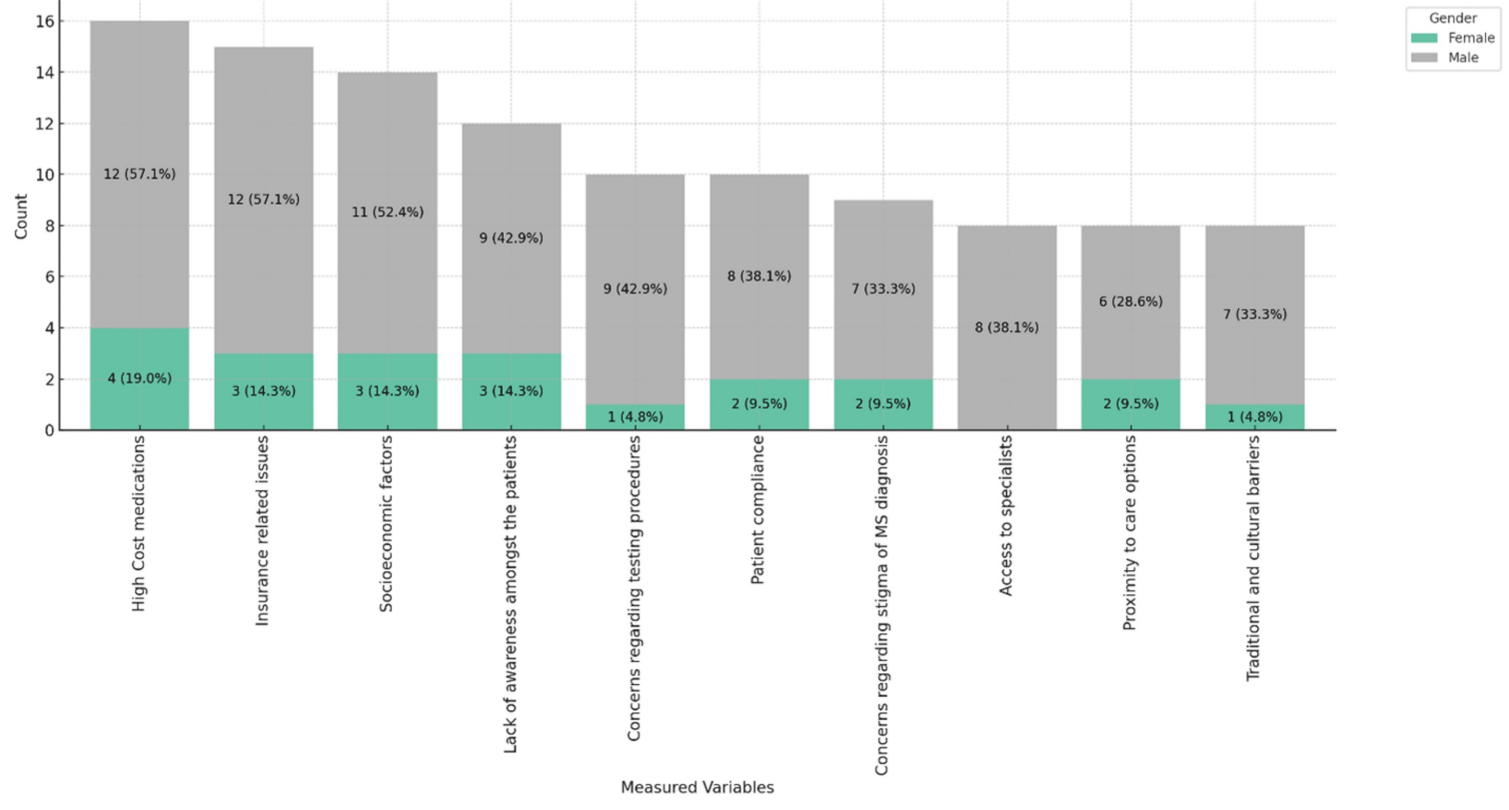
: Barriers physicians believe affect the care of MS patients in their practice, stacked by gender. MS: multiple sclerosis.

**Figure 13:**
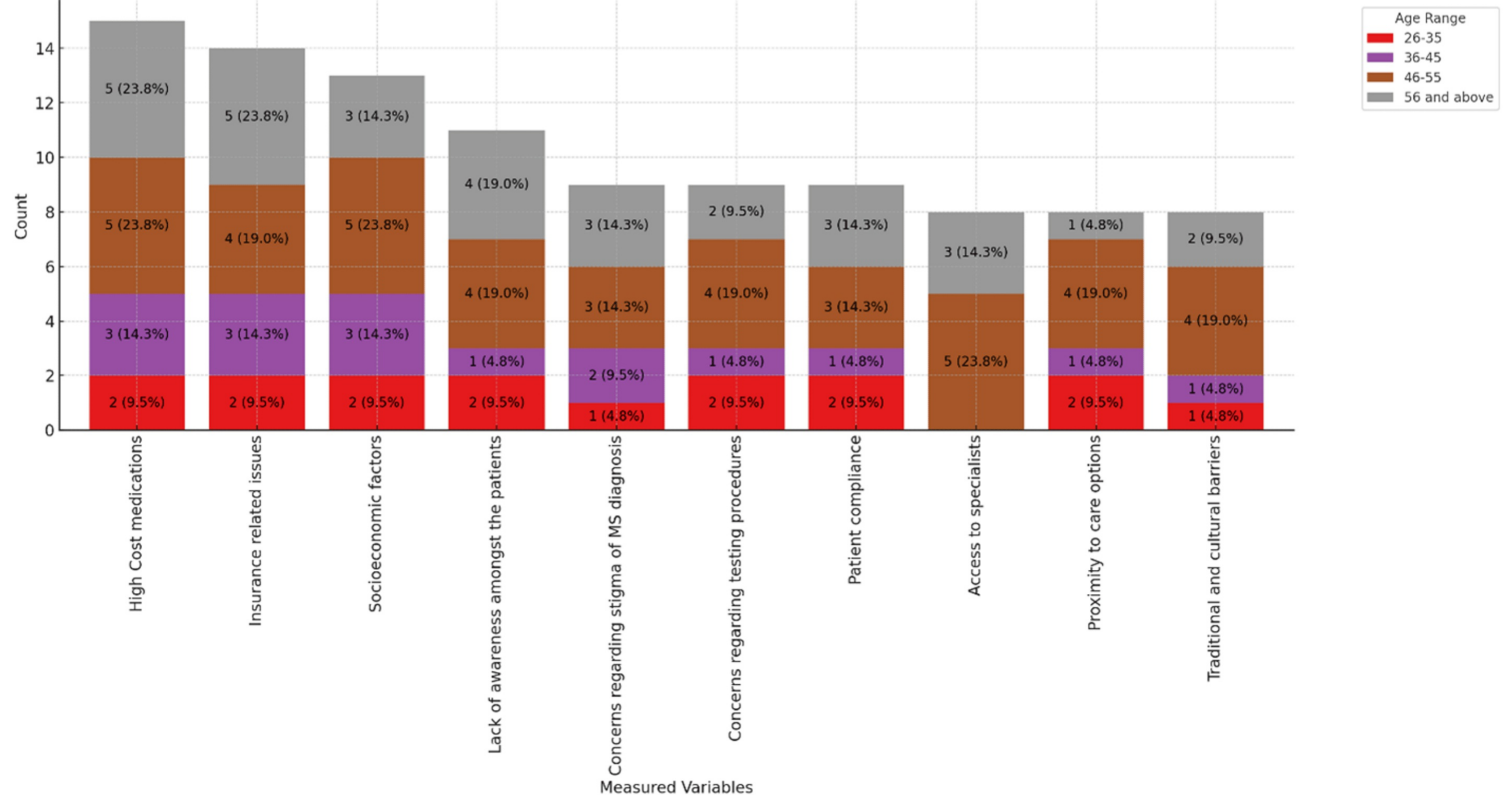
Barriers physicians believe affect the care of MS patients in their practice, stacked by age range. MS: multiple sclerosis.

Figures 11-13 provide insights into the barriers neurologists believe affect the care of MS patients in their practice, ranked in order of importance. Figure 11 shows that the high cost of medications (11, 52.4%) and insurance-related issues (10, 47.6%) are the most significant barriers, particularly in private practices. Socioeconomic factors (9, 42.9%) and patient compliance (7, 33.3%) are notable barriers. Figure 12 highlights that 12 (57.1%) male physicians report high medication costs and insurance issues as significant barriers, while for female physicians, these barriers are reported at 4 (19.0%) and 3 (14.3%), respectively. Figure 13 categorizes barriers by age range, showing a consistent concern across all age groups, particularly among those aged 46-55 and 56 and above. These figures underscore the multifaceted challenges in MS care, emphasizing financial and systemic barriers as the most critical issues across different demographics and practice settings.

## Discussion

The findings from this study highlight significant barriers to MS care in the UAE. The survey revealed that despite perceived high awareness of quality improvement tools, practical barriers persist, impacting the delivery of optimal MS care. This discussion will explore these barriers in detail, contextualize them with existing literature, and provide recommendations for future improvements.

The high awareness and use of quality improvement tools among respondents (18, 85.71%) is promising but may require further training and development of regional quality improvement tools. However, integrating these tools into everyday practice remains challenging. Previous studies have shown that while healthcare providers may be aware of quality improvement strategies, systemic and operational barriers often hinder their effective implementation [12].

MENACTRIMS was included in the quality improvement tool question to understand the neurologists' perspectives on using quality improvement tools in their practice. However, no specific published MENACTRIMS quality improvement tool has been mentioned in the literature. This indicates a potential gap in the availability of standardized quality improvement tools specific to the region, highlighting the need for developing and disseminating such tools tailored to local practice environments.

Regular measurement of clinical variables, such as screening for disease-modifying treatment side effects and MRI comparisons, is critical for effective MS management. Our study found that these practices are widely followed, with no significant variation across different practice settings. This aligns with the global standards for MS care, where regular monitoring is emphasized to manage disease progression [13].

However, it is noteworthy that some doctors do not usually perform the full EDSS assessment, which includes functional scores. This partial assessment may lead to an incomplete understanding of the patient's condition and potentially impact the effectiveness of the management plan. Ensuring that all components of the EDSS assessment are consistently utilized is crucial for a comprehensive evaluation of MS patients and for tailoring treatment plans more accurately.

Patient-related barriers, particularly insurance issues and the cost of medications, were frequently reported. These financial barriers significantly impact patient adherence to treatment plans. Similar findings were reported in other regions, underscoring the universal challenge of healthcare affordability in chronic disease management [14]. The high cost of MS treatments necessitates policy interventions to improve insurance coverage and reduce out-of-pocket expenses for patients.

Clinical practice barriers, such as insurance constraints, costs, and patient compliance issues, were prevalent. These barriers reflect broader systemic problems within the healthcare system. Studies have shown that reducing these barriers requires comprehensive strategies, including policy changes and enhanced patient support systems [15]. Addressing these issues can lead to better health outcomes and more efficient use of healthcare resources.

Physician-related barriers, including time constraints and lack of specialist support, were significant. Gender-related differences were noted, with female physicians reporting different challenges than their male counterparts. This finding suggests the need for tailored interventions that address the specific needs of diverse physician groups to enhance their capacity to provide high-quality MS care [16].

Healthcare system-related barriers, such as insurance provider policies and guideline coverage gaps, were prominent. These systemic issues hinder the delivery of consistent and effective MS care. Policy reforms to standardize insurance coverage and update clinical guidelines to cover all essential treatments are crucial to overcoming these barriers for MS patients [17].

Social tools identified as effective for improving MS care included increased community awareness and personalized support systems. These tools can enhance patient engagement and support and address social and psychological barriers. Implementing these tools requires collaborative efforts between healthcare providers, policymakers, and community organizations [18].

Comparative analysis with similar studies from other regions indicates that many barriers identified in the UAE are consistent with global challenges in MS care. However, some unique regional factors, such as cultural and educational barriers, require tailored strategies. Understanding these unique factors is essential for developing effective, context-specific interventions [19].

This study's limitations include the relatively small sample size and potential biases due to the survey method. These limitations suggest that the findings should be interpreted cautiously and that further research with larger, more diverse samples is needed to validate these results [20].

Based on the findings, several recommendations can be made. Enhancing insurance coverage, reducing treatment costs, and implementing targeted support for physicians are critical. Future research should focus on longitudinal studies to track the impact of these interventions and explore additional strategies to overcome identified barriers [21].

## Conclusions

Identifying and addressing the barriers to MS care in the UAE requires a multifaceted approach involving policy changes, systemic improvements, and enhanced support for both patients and healthcare providers. With the progressive improvement in the management of MS care, all stakeholders should be flexible in tackling these challenges to further enhance the quality of MS care and patient outcomes in the region. A task force should be set up to develop standardized quality improvement tools specific to the region and disseminate such tools tailored to local practice environments.

## Appendices

                                                                                         **Survey Questions**

1.      Do you consent to participate in this survey?

·         Yes

·         No

2.      What is your age range?

·         46-55

·         56 and above

·         36-45

·         26-35

3.      What is your gender?

·         Male

·         Female

4.      What is the place of your practice?

·         Private

·         Government

·         Semi-government

5.      Do you know any tools to measure quality improvement in MS care?

·         Yes

·         No

6.      Which variables do you measure regularly in your practice every 6-12 months? (Check all that apply)

·         EDSS assessment, at least yearly

·         Screening for Disease Modifying Treatment side effects

·         Monitoring and Comparison of MRI at least every 12 months

·         Cognitive screening

·         Bladder and Bowel screening

·         Exercise and approved Physical Activity counseling

·         Fatigue risk screening

·         Bladder and Bowel follow-up

·         Cognitive screening follow-up

·         Exercise and physical activity follow-up

·         Sexual dysfunction screening

·         Sexual dysfunction follow-up

·         All of the above

·         None of the above

·         Other (Specify)

7.      Specify the quality measurement tools or benchmarks used in your current practice if you answered "yes" to question 6. Choose one.

·         MENACTRIMS

·         American Association of Neurologists

·         NICE guidelines

·         Other (Specify)

8.      Do you advise your patients on any of the following regularly? (Select all that apply)

·         Vitamin D level monitoring

·         Psychological support

·         Smoking cessation

·         Blood pressure management

·         Weight management

·         Glycemic monitoring

·         None of the above

9.      Which of the following physician factors contribute to barriers in MS patient care? (Tick all that apply)

·         Insurance constraints

·         Time constraints

·         Lack of trained specialist nursing support

·         Lack of easy direct access to multidisciplinary team for MS care

·         Lack of training

·         Other (Specify)
